# Fragile X-Associated Tremor/Ataxia Syndrome: From Molecular Pathogenesis to Development of Therapeutics

**DOI:** 10.3389/fncel.2017.00128

**Published:** 2017-05-05

**Authors:** Ha Eun Kong, Juan Zhao, Shunliang Xu, Peng Jin, Yan Jin

**Affiliations:** ^1^Department of Human Genetics, School of Medicine, Emory UniversityAtlanta, GA, USA; ^2^The State Key Laboratory of Medical Genetics, School of Life Sciences, Central South UniversityChangsha, China; ^3^Department of Neurology, 2nd Hospital of Shandong UniversityJinan, China; ^4^Department of Ophthalmology, Second Hospital, Jilin UniversityChangchun, China

**Keywords:** FXTAS pathogenesis, CGG repeat, RNA toxicity, RAN translation, FXTAS therapeutics

## Abstract

Fragile X-associated tremor/ataxia syndrome (FXTAS) is a neurodegenerative disorder caused by a premutation CGG repeat expansion (55–200 repeats) within the 5′ UTR of the fragile X gene (*FMR1*). FXTAS is characterized by intension tremor, cerebellar ataxia, progressive neurodegeneration, parkinsonism and cognitive decline. The development of transgenic mouse and *Drosophila melanogaster* models carrying an expanded CGG repeat has yielded valuable insight into the pathophysiology of FXTAS. To date, we know of two main molecular mechanisms of this disorder: (1) a toxic gain of function of the expanded CGG-repeat *FMR1* mRNA, which results in the binding/sequestration of the CGG-binding proteins; and (2) CGG repeat-associated non-AUG-initiated (RAN) translation, which generates a polyglycine peptide toxic to cells. Besides these CGG-mediated mechanisms, recent studies have shed light on additional mechanisms of pathogenesis, such as the antisense transcript *ASFMR1*, mitochondrial dysfunction, DNA damage from R-loop formation and 5-hydroxymethylcytosine (5hmC)-mediated epigenetic modulation. Here we summarize the recent progress towards understanding the etiology of FXTAS and provide an overview of potential treatment strategies.

## Introduction

Fragile X-associated tremor/ataxia syndrome (FXTAS) is a neurodegenerative disorder caused by a CGG triplet repeat expansion within the 5′ UTR of *FMR1*. Normally, individuals possess between 5 and 54 CGG repeats, and full mutation CGG repeats greater than 200 lead to the neurodevelopmental disease fragile X syndrome (FXS), which results from the excessive methylation of *FMR1* and loss of FMRP protein (Kremer et al., 1991; Verkerk et al., 1991; Hagerman and Hagerman, 2002; Colak et al., 2014). Individuals with 55–200 CGG repeats are referred to as premutation carriers (Cronister et al., 2008).

Over a third of male expanded CGG repeat premutation carriers develop FXTAS later in adulthood (Jacquemont et al., 2004), whereas female premutation carriers may develop fragile X-associated primary ovarian insufficiency (FXPOI; Rodriguez-Revenga et al., 2009). Random X-inactivation is believed to protect female carriers from developing FXTAS, leading to relatively few female FXTAS patients (Hagerman et al., 2004; Zühlke et al., 2004; Coffey et al., 2008).

Clinically, FXTAS presents with intention tremor, gait ataxia, and other features including parkinsonism, cognitive defects, brain atrophy and white matter abnormalities on MRI (Jacquemont et al., 2003; Hagerman and Hagerman, 2015). Neuropathologically, FXTAS is distinguished by the characteristic ubiquitin-positive intranuclear inclusions in the brain and spinal cord as well as peripheral tissues (Greco et al., 2002, 2006; Gokden et al., 2009; Hunsaker et al., 2011).

Animal models have played a critical role in revealing the mechanisms of FXTAS pathogenesis. FXTAS mouse and *Drosophila melanogaster* models effectively mimic the molecular and cellular alterations and clinical symptoms of FXTAS. Several knock-in and transgenic mouse models are available for studying various aspects of FXTAS pathology (Bontekoe et al., 2001; Peier and Nelson, 2002; Entezam et al., 2007; Hashem et al., 2009). Aside from obviously elevated *FMR1* mRNA levels, reduced FMRP expression, and intranuclear inclusion formation, mouse models of FXTAS also exhibit abnormal dendritic spine morphology, impaired motor coordination, and cognitive deficits, recapitulating many features of FXTAS patients (Bontekoe et al., 2001; Willemsen et al., 2003; Entezam et al., 2007; Hunsaker et al., 2009; Hukema et al., 2015). In flies, the FXTAS transgenic *Drosophila* model expressing 90 CGG repeats displays locomotor deficits and retinal degeneration (Jin et al., 2003). Animal models allow researchers to investigate pathological mechanisms of FXTAS, identify potential modifiers, and pursue treatment development.

The two widely accepted mechanisms for the pathogenesis of FXTAS are RNA toxicity and repeat associated non-AUG translation (RAN) protein toxicity (via RAN). Several lines of evidence support the RNA toxicity mechanism. First, older adults with the full mutation ( >200 repeats), who do not express *FMR1* mRNA and lack FMRP, do not develop FXTAS (Feng et al., 1995). Second, in FXTAS, there is significant upregulation (2–8 fold) of the expanded CGG-repeat *FMR1* mRNA, resulting in formation of nuclear RNA aggregates. These aggregates sequester rCGG-binding proteins, preventing them from performing their normal biological functions, such as mRNA transcription and splicing, as well as dendritic mRNA transport (Tassone et al., 2000; Kenneson et al., 2001; Pretto et al., 2014). The level of FMR1 protein in cells from premutation carriers, however, remains relatively unaltered (Tassone et al., 2000; Kenneson et al., 2001). Third, *FMR1* RNA is present in the intranuclear inclusions of postmortem FXTAS brain tissue (Tassone et al., 2004), and animal and cell models expressing rCGG repeats develop similar inclusions (Jin et al., 2003; Willemsen et al., 2003; Arocena et al., 2005). But RNA toxicity alone is not sufficient to account for the large ubiquitin-positive intranuclear inclusions in the brains of FXTAS patients, a neuropathological hallmark of the disease. In fact, in addition to the RNA-binding proteins (RBPs), these inclusions contain proteins that do not bind to CGG-repeat mRNA and are reminiscent of the neuronal intranuclear inclusions found in protein-mediated neurodegenerative disorders and polyglutamine diseases (Greco et al., 2006; Iwahashi et al., 2006; Williams and Paulson, 2008). In light of this, a protein-driven mechanism of FXTAS pathogenesis was uncovered, in which the premutation CGG repeat expansion was found to induce RAN translation within the 5′ UTR of *FMR1* mRNA via an AUG-independent mechanism (Todd et al., 2013). The resulting polyglycine-containing protein, FMRpolyG, is present in the brains of FXTAS patients and was found to be toxic to human cell lines as well as *Drosophila* neurons, leading to retinal degeneration in FXTAS *Drosophila* (Todd et al., 2013).

To date, CGG repeat-mediated RNA toxicity and RAN protein toxicity stand as the two most important mechanisms in FXTAS pathophysiology, leading to the sequestration of specific proteins and the generation of the toxic protein product FMRpolyG, respectively. Besides these two main mechanisms, others have been uncovered, such as antisense *FMR1* RNA (Ladd et al., 2007), epigenetic modulation, mitochondrial dysfunctions (Hukema et al., 2014) and R-loop-induced DNA damage response (Loomis et al., 2014). In this review article, we summarize the current understanding of the underlying mechanisms of FXTAS and discuss potential therapeutic strategies.

## RNA-Mediated FXTAS Pathogenesis Via RBP Sequestration

A defining molecular signature of FXTAS is the elevation of premutation *FMR1* mRNA levels with no detectable or only a modest reduction in FMRP protein levels (Tassone et al., 2000; Kenneson et al., 2001; Pretto et al., 2014). Along with the presence of *FMR1* mRNA in ubiquitin-positive intranuclear inclusions of FXTAS patient brains, these observations point to a toxic RNA gain-of-function mechanism for FXTAS pathogenesis, which could lead to sequestration of various rCGG repeat-binding proteins (Tassone et al., 2004).

Using mass spectrometric analysis combined with immunohistochemical analysis, more than 20 proteins have been identified in inclusions in the frontal cortex of FXTAS patients, including RBPs hnRNP A2/B1 and MBNL1, and some neurofilament proteins, such as lamin A/C and α-internexin, which are involved in various neurological disorders (Iwahashi et al., 2006). Pur α and hnRNP A2/B1 are found to bind directly to rCGG repeats in inclusions. In fact, in a *Drosophila* model expressing premutation CGG repeat expansions, overexpression of Pur α and hnRNP A2/B1 leads to suppression of neurodegeneration phenotypes (Jin et al., 2007; Sofola et al., 2007). Sequestration of other proteins, such as CUGBP1, Sam68, Rm62 and DGCR8, leads to altered mRNA splicing and transport, as well as dysregulated microRNAs (Sofola et al., 2007; Sellier et al., 2010, 2013; Qurashi et al., 2011; Tan et al., 2012a). These findings support a toxic RNA gain-of-function mechanism, which is mediated by the expanded CGG repeats in *FMR1*.

Heterogeneous nuclear ribonucleoprotein (hnRNP A2/B1) is an RBP noted for its presence in intranuclear inclusions of FXTAS patients. hnRNP A2/B1 has been shown to bind directly to rCGG repeats and, interestingly, overexpression of hnRNP A2/B1 and its two homologs in *Drosophila* results in suppression of the neurodegenerative eye phenotype caused by the rCGG repeat (Sofola et al., 2007). hnRNP A2/B1 is also known to mediate the indirect interaction between CGG repeats and CUGBP1, a RBP noted for its binding of CUG repeats and involvement in myotonic dystrophy type 1 (DM1; Timchenko et al., 1996, 2001, 2004). Overexpression of CUGBP1 also suppresses the FXTAS phenotype in *Drosophila* (Sofola et al., 2007). Moreover, Muslimov et al. (2011) found that premutation CGG-repeat RNA binds competitively to hnRNP A2/B1 and leads to the alteration of neuronal RNA dendritic transport, which can be partially reversed by overexpression of hnRNP A2. Furthermore, our group has shown that in a *Drosophila* model, rCGG repeats trigger the activation of certain retrotransposons, such as *gypsy*. We also demonstrated that hnRNP A2/B1 may regulate the activation of *gypsy* by recruiting heterochromatin protein 1 (HP1) for transposon silencing. As a result, in FXTAS, the expanded rCGG repeats may sequester hnRNP A2/B1 and diminish the recruitment of HP1 to genomic regions containing retrotransposons, further contributing to retrotransposon activation (Tan et al., 2012b). Significantly, we also found that hnRNP A2/B1 regulates expression of miR-277, a miRNA that, when overexpressed, results in enhancement of rCGG repeat-mediated neurodegeneration (Tan et al., 2012a).

Also present in intranuclear inclusions of FXTAS patients, Pur α is an RNA- and specific single-stranded DNA-binding protein that plays an essential role in DNA replication, neuronal mRNA transport, and translation. Pur α knockout mice display developmental delay along with severe tremor and spontaneous seizures at 2 weeks after birth, and the expression and distribution of axonal and dendritic proteins are also altered (Khalili et al., 2003; Hokkanen et al., 2012). Similar to hnRNP A2/B1, Pur α has been implicated in FXTAS pathogenesis due to the fact that overexpression of Pur α in a *Drosophila* model results in dose-dependent suppression of rCGG-mediated neurodegeneration.

Probing the Pur α interactome has also led to the identification of Rm62 as a possible mediator of FXTAS pathogenesis. Rm62 was identified via a proteomic approach as a potential regulator of rCGG-mediated neurodegeneration (Qurashi et al., 2011). Rm62 is the *Drosophila* ortholog of p68 RNA helicase, a transcriptional regulator that is also involved in pre-mRNA splicing, RNA interference and nucleocytoplasmic shuttling (Bond et al., 2001; Ishizuka et al., 2002; Liu, 2002; Wilson et al., 2004; Lin et al., 2005). In FXTAS *Drosophila*, rCGG repeats diminish posttranscriptional expression of Rm62, and overexpression of Rm62 can suppress the neuronal toxicity caused by the premutation rCGG repeats (Qurashi et al., 2011). The decrease in Rm62 expression in turn leads to the nuclear accumulation of mRNAs involved in stress and immune responses, as well as the accumulation of *Hsp70* mRNA, a target of Rm62 (Qurashi et al., 2011), which has actually been found in inclusions from both human FXTAS brains and animal models of FXTAS (Jin et al., 2003).

The alternative splicing regulator Src-Associated substrate during mitosis of 68-kDa (Sam68) is another RBP shown to be sequestered by rCGG repeats. One study revealed that Sam68 colocalizes with the giant dynamic aggregates that form from mRNAs containing expanded CGG repeats in both premutation CGG-expressing cells and FXTAS patient brain sections (Sellier et al., 2010). The sequestration of Sam68 results in the loss of its ability to perform splicing regulation and causes pre-mRNA alternative splicing misregulation in CGG-transfected cells and FXTAS patients (Sellier et al., 2010). Sam68 localization is regulated by tyrosine phosphorylation, and the phosphatase inhibitor tautomycin was shown to prevent aggregation of both Sam68 and CGG RNA (Sellier et al., 2010). Taken together, the sequestration of Sam68 must play a role in FXTAS pathogenesis via a splicing alteration mechanism.

TAR DNA-binding protein (TDP-43) is an amyotrophic lateral sclerosis (ALS)-associated RBP, a marker of neurodegeneration commonly found in inclusions in ALS (Baloh, 2012). In the cerebellar Purkinje neurons of mice expressing 90 CGG repeats, the mRNA for *Tardbp*, which encodes TDP-43, showed a reduced association with ribosomes (Galloway et al., 2014). In the same study, the authors went on to find that in the *Drosophila* model of FXTAS, wild-type TDP-43 expression leads to suppression of neurodegeneration, while knockdown of the endogenous TDP-43 fly ortholog, TBPH, enhanced the eye phenotype. Another study also independently reported that TDP-43 suppresses CGG repeat-induced toxicity in a *Drosophila* model of FXTAS (He et al., 2014). Interestingly, this suppression was shown to depend on hnRNP A2/B1, such that deletion of the C-terminal domain of TDP-43 and thereby the prevention of interactions with hnRNP A2/B1 led to abrogation of the TDP-43-dependent rescue of CGG repeat toxicity (He et al., 2014).

The DiGeorge syndrome critical region 8 (DGCR8) is yet another protein reported to bind to premutation rCGG repeats and cause partial sequestration of DGCR8 and its partner, DROSHA, within the premutation RNA aggregates (Sellier et al., 2013). DGCR8 and DROSHA play a critical role in microRNA biogenesis. In the first step of microRNA biogenesis, RNA polymerase II transcribes miRNAs as primary miRNA (pri-miRNA) transcripts. DROSHA, a type III RNase, is anchored to the pri-miRNA by DGCR8, and processes pri-miRNA into precursor miRNA (pre-miRNA; Lee et al., 2003; Denli et al., 2004; Gregory et al., 2004; Han et al., 2004; Landthaler et al., 2004). Sellier et al. (2013) found that the sequestration of DGCR8 and DROSHA precludes them from their normal functions, leading to reduced processing of pri-miRNAs in cells expressing expanded CGG repeats and also in the brains of FXTAS patients. Consequently, levels of mature miRNAs are reduced (Sellier et al., 2013). The authors also found evidence that sequestration of SAM68 in the CGG aggregates is mediated through DROSHA or DGCR8, but restoration of SAM68 function is not sufficient to restore all normal neuronal cell functions. In contrast, expression of DGCR8 alone in cultured premutation mouse cortical neurons could rescue their dendritic morphological abnormalities and diminished neuronal viability. This work suggested a model for the mechanism of FXTAS pathogenesis in which sequestration of the DROSHA-DGCR8 microprocessor by expanded rCGG repeats may lead to reduced mature miRNA expression, resulting in neuronal cell dysfunction and degeneration.

**Figure 1:**
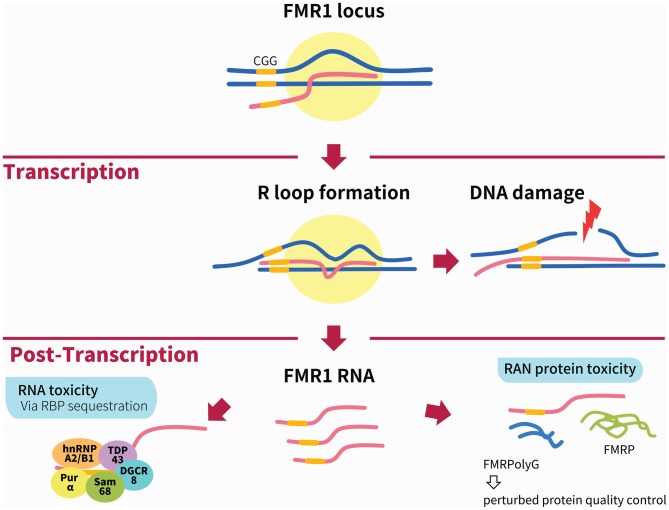
**Illustration of the main mechanisms of Fragile X-associated tremor/ataxia syndrome (FXTAS) pathogenesis.** During transcription of the* FMR1* locus, the formation of RNA:DNA hybrid R-loops through GC interaction of the expanded CGG repeats (depicted by yellow bar) can activate the DNA damage response and result in DNA breaks and the accumulation of γH2AX. The two main mechanisms linked to FXTAS pathology are post-transcriptional. In the RNA toxicity mechanism, RNA-binding proteins (RBPs) are sequestered by the expanded CGG repeats, such as h2RNP A2/B1, Pur α, Sam68, TDP43 and DGCR8; these are illustrated together for figurative purposes but do not necessarily form a complex simultaneously. In the RAN protein toxicity mechanism, the expanded CGG repeat induces AUG-independent RAN translation of FMRpolyG, which is found to form inclusions in patient brains as well as animal models of FXTAS. Other mechanisms not shown in this figure include the antisense transcript *ASFMR1*, mitochondrial dysfunction and 5hmC-mediated epigenetic modulation in FXTAS.

In summary, we have presented a broad overview of the RBPs that are sequestered via the RNA toxicity mechanism, as well as our current limited understanding of the downstream effects of sequestration (Figure 1). The challenge remains to bring the pieces of the puzzle together to figure out how the sequestration of the RBPs interplay to cause FXTAS pathology (Hagerman, 2012).

## RAN Translation in FXTAS Pathogenesis

In FXTAS pathogenesis, RAN protein toxicity plays a synergistic role with the RNA toxicity mechanism and offers a potential explanation for the presence of non-RBPs in the distinctive ubiquitin-positive intranuclear inclusions found in the brains of FXTAS patients. First discovered in CAG expansion constructs, RAN translation initiates in an AUG-independent manner and is known to occur in several repeat expansion disorders, among them Spinocerebellar Ataxia type 8 (SCA8), DM1 Frontotemporal Dementia (FTD), and ALS, as well as FXTAS (Zu et al., 2011, 2013; Ash et al., 2013; Mori et al., 2013). RAN translation initiation requires an m^7^G cap, the EIF4A helicase and 40S ribosomal scanning and is strongly influenced by repeat length (Kearse et al., 2016). In CGG-induced RAN translation of *FMR1* mRNA, initiation of translation is similar to canonical translation but only 30%–40% as efficient. The expanded premutation CGG repeat expansion induces AUG-independent RAN translation of FMRpolyG, which accumulates in the ubiquitin-positive intranuclear inclusions in transfected cells, FXTAS *Drosophila*, mouse models and patient brains (Todd et al., 2013). Out of three possible reading frames in the 5′ UTR of *FMR1*, RAN translation occurs in both the glycine (+1 frame) and alanine (+2 frame) reading frames, producing the FMRpolyglycine and FMRpolyalanine proteins, respectively, with no polyarginine product (+0 frame) detected as yet (Todd et al., 2013). However, only the polyglycine-containing protein, FMRpolyG, is detected in both cultured cells and animal models of FXTAS, and most importantly, in inclusions in FXTAS patient brains (Todd et al., 2013). Moreover, the expression of FMRpolyG is known to be toxic to *Drosophila* neurons, leading to retinal degeneration that can be enhanced by increasing FMRpolyG product and suppressed by eliminating RAN translation (Todd et al., 2013).

To determine the mechanism by which FMRpolyG production may contribute to FXTAS pathogenesis, Oh et al. (2015) examined ubiquitin-proteasome system (UPS) impairment in *Drosophila* and cell models of CGG repeat-induced toxicity. UPS impairment in FXTAS *Drosophila* led to enhanced neurodegeneration, whereas overexpression of HSP70 suppressed this toxicity. Furthermore, in cell models, the expression of FMRpolyG increased induction of UPS impairment, whereas prevention of RAN translation resulted in diminished UPS impairment (Oh et al., 2015). These findings indicate that RAN translation-induced FMRpolyG production may drive FXTAS pathogenesis by perturbing the protein quality control pathway through UPS failure (Oh et al., 2015).

Recently, researchers found that RAN translation also occurs from antisense *FMR1* transcript containing CCG repeats from three different potential reading frames generating polyproline, polyarginine and polyalanine proteins. More importantly, these novel proteins are found to colocalize with ubiquitinated intranuclear inclusions in FXTAS patient neurons. These new findings provide additional support for RAN protein toxicity in FXTAS pathophysiology (Krans et al., 2016).

## Other Mechanisms of FXTAS Pathogenesis

### R-Loop-Mediated DNA Damage

In addition to post-transcriptional changes, there is evidence to suggest that the increase in FXTAS *FMR1* RNA transcript results in molecular dysfunction at the transcriptional level. Loomis et al. (2014) have found that, upon transcription of the endogenous *FMR1* locus, the nascent G-rich RNA transcribed from the GC-rich region of *FMR1* 5′ UTR binds to the C-rich DNA template and forms a stable RNA: DNA hybrid, or R-loops (Reddy et al., 2011; Loomis et al., 2014). Notably, R-loop formation can be increased by enhanced transcription (Loomis et al., 2014). Excessive R-loop formation activates the DNA damage response and leads to DNA breaks, resulting in accumulation of γH2AX, a histone variant associated with DNA damage repair that is also present in the inclusions of FXTAS patient neurons (Iwahashi et al., 2006; Hoem et al., 2011). These results clearly show that FXTAS pathology from the RNA toxicity mechanism may also occur co-transcriptionally via increased R-loop formation upon increased *FMR1* transcription, leading to subsequent DNA damage.

### Antisense Transcript: ASFMR1

A lasting conundrum in the pathogenesis of FXTAS is that only a fraction of male premutation carriers develop FXTAS (Jacquemont et al., 2004), and there is considerable variability in the phenotype of FXTAS patients. Such observations raise the likelihood that *FMR1* is not the sole gene responsible for FXTAS pathogenesis. In the search for alternate gene involvement, Ladd et al. (2007) identified an antisense transcript spanning the *FMR1* CGG repeat region in the antisense direction, which they dubbed the antisense transcript at the *FMR1* locus, *ASFMR1*. Congruous to *FMR1, ASFMR1* is upregulated in premutation carriers but silenced in the full-mutation range. Following transcription, *ASFMR1* is spliced, and then transported to the cytoplasm. *ASFMR1* transcription is driven by two alternative promoters: the *FMR1* bidirectional promoter and the promoter in the second intron of *FMR1*. The latter is considered to be the major promoter in cells with premutation alleles and drives the transcription of the transcript, which spans the CGG repeat of the *FMR1* gene in the CCG orientation and exhibits premutation-specific alternative splicing (Ladd et al., 2007). These findings strongly suggest that *ASFRM1* is also implicated in the pathogenesis of FXTAS.

### Mitochondrial Dysfunction

There is also evidence to indicate that *FMR1* premutation-associated disorders, such as FXTAS and FXPOI, involve mitochondrial dysfunction. Using doxycycline (dox)-inducible transgenic mouse models expressing 90 CGGs in the RNA, Hukema et al. have found that dox-induced 90 CGG RNA-expressing mice not only experience loss of weight, death within 5 days, steatosis, and apoptosis in the liver, but that they also show altered expression of GPX1 and cytochrome C, markers of mitochondrial dysfunction (Hukema et al., 2014). Mitochondrial dysfunction is also seen in FXTAS human patients and mouse cultured cells. Along with decreased oxidative phosphorylation capacity, there is a defect in the import of mitochondrial proteins in premutation carriers (Napoli et al., 2011). Hippocampal neuronal cultures from premutation CGG knock-in mice exhibit mitochondrial abnormalities in the number, metabolic function and mobility of mitochondria (Kaplan et al., 2012). Mitochondrial abnormalities are also seen in granulosa cells and oocytes in a FXPOI mouse model, including reduced mitochondrial content, abnormal mitochondrial structure, and decreased expression of the mitochondrial genes *Mfn2* and *Opa1* (Conca Dioguardi et al., 2016). Hence, these findings point to mitochondrial dysfunction as another possible component of FXTAS pathology.

### 5-hydroxymethylcytosine (5hmC)-Mediated Epigenetic Modulation

Though initially regarded as only an intermediate of active DNA demethylation products (Pfaffeneder et al., 2011), 5-hydroxymethylcytosine (5hmC) has gained attention more recently as an epigenetic modification with a significant role in processes like neurodevelopment and differentiation (Branco et al., 2011). 5hmC is converted from 5mC via catalysis by Ten-eleven translocation 1 (TET1), a 2-oxoglutarate (2OG)- and Fe (II)-dependent enzyme (Tahiliani et al., 2009). Our group has examined the global levels of 5hmC in the cerebella of rCGG mice compared to wild-type age-matched littermate controls (Yao et al., 2014). Significantly, 5hmC was reduced genome-wide in the cerebella of the rCGG mice, while several repetitive elements as well as cerebellum-specific enhancers exhibited increases in 5hmC levels. The differential 5-hydroxymethylated regions (DhMRs) were highly correlated with genes and transcription factors that play key roles in neuronal development. Furthermore, Esanov et al. (2016) have found that there is increased hydroxymethylation at the FMR1 promoter in the brains of FXS full-mutation patients compared to premutation carriers and unaffected controls. While these data support 5hmC-mediated epigenetic modulation as a player in FXTAS pathogenesis, further studies, such as characterizing the level of 5hmC at the *FMR1* locus, are warranted to reveal more about the role of the 5hmC mark in the pathogenic mechanism of FXTAS (Al-Mahdawi et al., 2014).

## Therapeutic Developments

**Figure 2:**
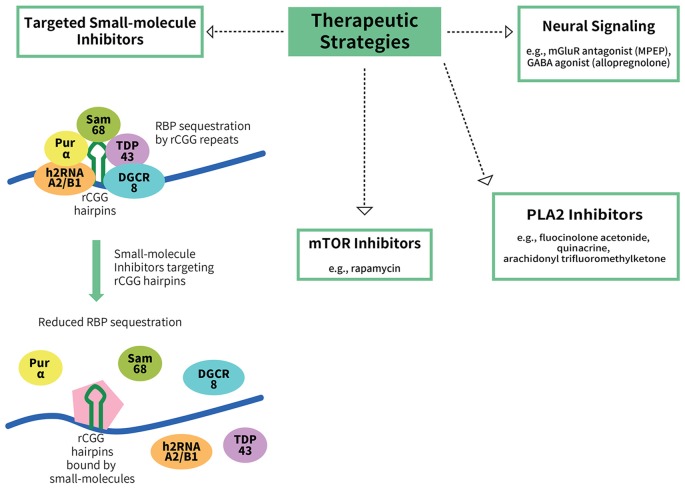
**Potential therapeutic strategies for FXTAS.** To date, there are no treatments available for FXTAS. Therapeutic strategies explored include targeting the RBP sequestration mechanism of FXTAS pathogenesis, such as via the use of small-molecule inhibitors that bind to CGG hairpins with high affinity, thereby reducing the sequestration of the RBPs (Disney et al., 2012). Other potential targets include the mTOR inhibitor rapamycin, phospholipase A2 (PLA2) inhibitors, an mGluR antagonist (MPEP) and a GABA agonist (allopregnanolone).

As there are still no effective treatment options for FXTAS, current therapeutic strategies are limited mainly to treatments aimed at ameliorating specific symptoms of FXTAS patients. Selective serotonin and norepinephrine reuptake inhibitors have been effective for anxiety and depression (Hagerman and Hagerman, 2015). Psychosis and tremor can be improved by atypical antipsychotics, such as propranolol and primidone (Zesiewicz et al., 2005; Muzar and Lozano, 2014). Although the NMDA receptor antagonist memantine, an FDA-approved compound for the treatment of Alzheimer’s disease, did not show any significant improvement on tremors, balance problems, or executive function deficits in FXTAS patients compared to placebo (Seritan et al., 2014), verbal memory was improved when assessed by the event-related potential (ERP) paradigm in a subgroup of patients in a recent clinical trial (Yang J. C. et al., 2016). At the same time, however, the end goal is not mere symptomatic treatment, but rather effective treatments that target the pathogenic mechanism(s) of disease. Here we discuss some ongoing efforts to identify potential compounds and therapeutic targets in FXTAS both *in vitro* and *in vivo* (Figure 2).

MPEP and allopregnanolone, an mGluR antagonist and a GABA agonist, respectively, are potential therapeutics that may mitigate some of the RNA toxicity effects. Cortical astrocytes from mice expressing premutation CGG repeats display decreased expression of Glu transporters (GLT-1 and GLAST) and Glu uptake, and enhanced spontaneous asynchronous Ca^2+^ oscillations (Cao et al., 2013). An mGluR5 antagonist, 2-methyl-6-(phenylethynyl)pyridine hydrochloride (MPEP), suppresses the intracellular Ca^2+^ increase induced by Glu (Cao et al., 2012, 2013). Although MPEP is neurotoxic to humans and cannot be used for therapy, these results shed light on Glu transport and Ca^2+^ signaling as potential targets for treatment. The natural neurosteroid allopregnanolone is a GABA signaling agonist, and it was found to ameliorate the clustered burst firing in hippocampal neurons from mice with *FMR1* premutation alleles (Cao et al., 2012; Reddy and Rogawski, 2012). Allopregnanolone exhibits effective rescue in Alzheimer’s disease mouse models and human cells, such as restoring learning and memory, in addition to improving neuronal proliferation and survival (Wang et al., 2010; Chen et al., 2011; Singh et al., 2012). Allopregnanolone is already in use in Alzheimer’s disease and traumatic brain injury clinical trials, making it a very likely treatment choice for FXTAS (Lozano et al., 2015).

In a chemical screen of efficient small molecules to suppress locomotion deficits and neurodegeneration of FXTAS *Drosophila*, several phospholipase A2 (PLA2) inhibitors were found to have significant effects, namely fluocinolone acetonide, quinacrine and arachidonyl trifluoromethyl ketone. These findings suggest a role for altered PLA2 activity in FXTAS and present a potential therapeutic target (Qurashi et al., 2012). Another potential target is the mammalian target of rapamycin (mTOR). Although the mTOR inhibitor rapamycin was shown to suppress neurotoxicity via autophagy activation in various neurodegenerative disease animal models, it can also enhance the neurodegeneration phenotypes in FXTAS *Drosophila*, such as aggravation of retinal degeneration and locomotion defects, as well as a shorter lifespan of flies (Lin et al., 2013). In contrast, genetic activation of mTOR signaling significantly suppresses the neurodegeneration phenotype (Lin et al., 2013).

Although a number of therapeutic strategies are being explored that specifically target some of the underlying pathogenic mechanisms of FXTAS discussed above, as of now targeted therapeutic development is far from clinical trials. Small molecules are being developed with a high affinity for rCGG hairpins that inhibit target proteins from binding to rCGG hairpins *in vitro*, thereby reducing the sequestration of RBPs by rCGG (Disney et al., 2012). Some compounds, such as 9-hydroxy-5,11-dimethyl-2-(2-(piperidin-1-yl)ethyl)-6H-pyrido[4, 3-b]carbazol-2-ium, improve pre-mRNA splicing deficits and reduce the number and size of CGG protein aggregates in FXTAS cells (Disney et al., 2012; Tran et al., 2014). Moreover, most recently, Yang W. Y. et al. (2016) reported the generation of designer, modularly assembled small molecules that bind rCGG expanded repeats and potently improve FXTAS-associated defects in cells.

Finally, in addition to small molecules, the histone acetyltransferase (HAT) inhibitors garcinol and anacardic acid have also been pursued as potential therapeutics for FXTAS. In an effort to understand why there is *FMR1* mRNA buildup in FXTAS pathology, Todd et al. (2010) examined histone acetylation at the human *FMR1* locus and found, interestingly, that histone acetylation was increased at the *FMR1* locus in premutation carriers compared to control or FXS-derived cell lines, which correlated with increased *FMR1* mRNA levels in premutation cell lines. This study went on to show that in premutation carrier cell lines, the HAT inhibitors garcinol and anacardic acid can repress *FMR1* mRNA expression down to the level of control and can also extend the lifespan of *Drosophila* expressing the CGG repeat expansion (Todd et al., 2010). Based on these results, a novel mechanism was posited for the increased premutation *FMR1* mRNA in FXTAS pathology. According to the proposed model, the expanded CGG repeats in FXTAS induces chromatin remodeling in *cis*, which leads to increased expression of *FMR1* mRNA. This study provides the basis for a new potential therapeutic strategy for FXTAS by using HDACs to control the increased expression of *FMR1* mRNA-containing expanded CGG repeats.

Although it would be a challenge to bring these compounds to the bedside in the immediate future, development of these compounds enriches our understanding of therapeutic targets and extends the frontier of therapeutic development.

## Perspective and Future Directions

Over the past decade, there has been considerable progress in our understanding of FXTAS and its pathogenesis. Two potential mechanistic models to explain the molecular pathogenesis of FXTAS have taken root and begun to solidify; namely, the RNA toxicity and the RAN protein toxicity mechanisms. In addition, we are beginning to develop a much more holistic understanding of other players in FXTAS pathogenesis, such as *ASFMR1*, mitochondrial dysfunction, and the implications of the 5hmC epigenetic mark (Ladd et al., 2007; Napoli et al., 2011; Kaplan et al., 2012; Hukema et al., 2014; Conca Dioguardi et al., 2016). However, much of our understanding of FXTAS pathogenesis is incomplete, and many questions remain unanswered.

Although we have identified numerous players in FXTAS pathogenesis, we still lack enough mechanistic and chronological understanding to identify the best targets for treatment. Indeed, in terms of chronology, studies in mice have shown that premutation expanded CGG repeats in the mouse *Fmr1* gene perturb embryonic neocortical development (Cunningham et al., 2011). Also, in another study, cultured hippocampal neurons from mice expressing premutation CGG repeats exhibited shorter dendritic lengths and fewer branches between 7–21 days *in vitro* compared to wild-type littermates (Chen et al., 2010). These studies and others have raised the possibility that the onset of FXTAS may be the result of a lifelong pathologic process (Garcia-Arocena and Hagerman, 2010). If it is true that premutation carriers are predisposed to this lifelong pathologic process beginning in infancy, one of our next tasks would be to determine the factors that distinguish the premutation carriers who go on to develop FXTAS in late adulthood from those who are somehow protected. In addition, we would need to identify the earliest period for potential therapeutic intervention.

Variability in FXTAS is not limited to the onset of disease; there is also a broad spectrum of phenotypes (Garcia-Arocena and Hagerman, 2010). Although the main clinical manifestations remain locomotor in nature, FXTAS can also manifest as significant non-motor neurodegenerative phenotypes, such as cognitive decline/dementia and neuropsychiatric disturbances (Bourgeois et al., 2009; Garcia-Arocena and Hagerman, 2010). Adding to the phenotypic variability, in the low proportion of FXTAS patients who are female carriers, it is more common to see clinical features such as autoimmune-type dysfunction, hypothyroidism, and muscle pain than in men (Coffey et al., 2008; Garcia-Arocena and Hagerman, 2010). Thus, in addition to further decoding the molecular basis of FXTAS, subsequent efforts should be directed toward the identification of genetic modifiers that account for the variability in onset and phenotype seen with FXTAS.

## Author Contributions

HEK and JZ wrote the manuscript. SX edited it and provided the input. PJ and YJ edited the final version of the manuscript.

## Conflict of Interest Statement

The authors declare that the research was conducted in the absence of any commercial or financial relationships that could be construed as a potential conflict of interest.
